# RNA interference mediated pten knock-down inhibit the formation of polycystic ovary

**DOI:** 10.1007/s11010-013-1673-z

**Published:** 2013-05-19

**Authors:** Jie-Xiu Ouyang, Tao Luo, Hui-Yun Sun, Jian Huang, Dan-Feng Tang, Lei Wu, Yue-Hui Zheng, Li-Ping Zheng

**Affiliations:** 1 Medical Experimental Teaching Department, Nanchang University, Nanchang, 330031 China; 2 Institute of Life Science, Nanchang University, Nanchang, 330031 China; 3 Department of Physiology Reproduction, Medical College of Nanchang University, Nanchang, 330036 China

**Keywords:** Polycystic ovary, *pten*, RNA interference, Follicular development

## Abstract

*Pten* (phosphatase and tensin homolog deleted on chromosome 10), a kind of tumor suppressor gene, plays important roles in female reproductive system. But its expression and roles in the formation of polycystic ovaries are yet to be known. In this study, we constructed a rat model of PCOS using norethindrone and HCG injections and found the expressions of pten mRNA and PTEN protein increased significantly in the polycystic ovary tissue by immunohistochemistry, RT-PCR, and western blot. Furthermore, the results showed that in vivo ovaries could be effectively transfected by lentiviral vectors through the ovarian microinjection method and indicated that pten shRNA may inhibit the formation of polycystic ovaries by pten down-regulation. Our study provides new information regarding the role of PTEN in female reproductive disorders, such as polycystic ovary syndrome.

## Introduction

Polycystic ovary syndrome (PCOS), an endocrine disorder most common in women during their reproductive years, is characterized by at least two of the following: hyperandrogenism, oligo-anovulation, hirsutism, and polycystic ovaries [1–3]. In addition, affected women are often obese and insulin-resistant [4, 5] PCOS is known to be a major cause of female infertility.

The importance of PTEN (phosphatase and tensin homolog deleted on chromosome ten) is illustrated by its frequent mutation in cancers. By suppressing the phosphoinositide 3-kinase (PI3K)-AKT-mammalian target of the rapamycin (mTOR) pathway through its lipid phosphatase activity, PTEN governs a plethora of cellular processes, including survival, proliferation, energy metabolism, and cellular architecture [6]. Furthermore, it has been shown that a lack of PTEN in oocytes can cause premature ovarian failure in mice and that controlling the initiation of the oocyte growth PTEN-PI3K pathway can regulate the follicle activation [7]. PTEN has recently been characterized as a regulator of the survival and life span of granulosa/luteal cells, facilitated ovulation and the persistence of non-steroidogenic luteal structures [8]. PTEN expression may be a key to the regulation of function in human granulose cells as well as the pathogenesis of PCOS [9]. However, further studies are needed to explore whether *pten* expression is related to abnormal follicular development (e.g., early excessive follicular growth and impeded dominant follicular formation) in PCOS.

For this paper, we used an animal model of PCOS to study the expression of PTEN in polycystic ovaries and to investigate its role in the formation of polycystic ovaries. Our research will provide a better understanding of the pathogenic mechanisms involved in PCOS and of the molecular mechanisms necessary for the diagnosis of related female reproductive disorders.

## Materials and methods

### Animals and experimental protocols

The experimental design and animal care and use conformed to the National Institute of Health guidelines on the ethical use of animals. Twenty-four-day-old Sprague–Dawley (SD) female rats were purchased from the Medical Experimental Animal Center of Nanchang University. The rats were divided into the model group (*n* = 36) and the control group (*n* = 12) according to their age, size, and vitality. Rats in both groups received a subcutaneous implant of norethindrone. After three days, rats in the model and in the control groups were injected with HCG (1.5 IU/0.2 mL) and normal saline (0.2 mL), respectively, twice a day for the next 9 days [10].

The rats were killed by decapitation, and blood was collected to measure serum hormone levels. The serum was separated from the red blood cells by centrifugation and stored at 4 °C for subsequent assay. 1 week after killing the rats, the levels of sex hormones, including FSH, LH, *T* (testosterone), *E* (estradiol), and INS (insulin), were measured by electrochemiluminescence immunoassays. The ovaries were first removed and weighed and then fixed in 4 % formaldehyde and stained with HE (hematoxylin and eosin) for light microscopy. Immunohistochemical methods were used to compare the level of PTEN expression in the polycystic ovarian tissues of each group.

### RNA extraction and RT-PCR

Total RNA was extracted from the isolated ovary tissues of each rat with Trizol Reagent (Invitrogen). The integrity of the extracted total RNA was detected by 1 % agarose gel electrophoresis, and the RNA concentration was determined under ultraviolet (UV) light at 260 nm. RT-PCR was performed with a one-step RT-PCR kit (Qiangen) according to the manufacturer’s protocol, using 32 cycles on 2 ng total RNA. Primers for *pten* and *β*-*actin* (control) were as follows:

- *pten* forward: 5′-ACCATAACCCACCACAGC-3′;
- *pten* reverse: 5′-CTGCCTGACCACATTACTAA-3′;
- *β*-*actin* forward: 5′-TCAGGTCATCACTATCGGCAAT-3′;
- *β*-*actin* reverse: 5′-AAAGAAAGGGTGTAAAACGCA-3′;

The PCR products were loaded onto 2.5 % agarose gel and then visualized and quantified by the GeneGenius Gel Imaging System and Image Quanta Analysis Software (Syngene, American). The optical density ratio (OD pten/OD β-actin) was used to indicate the expression levels of *pten* mRNA.

### Western blot

Total protein was extracted from ovary tissues. Western blotting was performed according to the method proposed by Jiang et al. [11].

### Vectors for *pten shRNA*

**Fig. 1:**
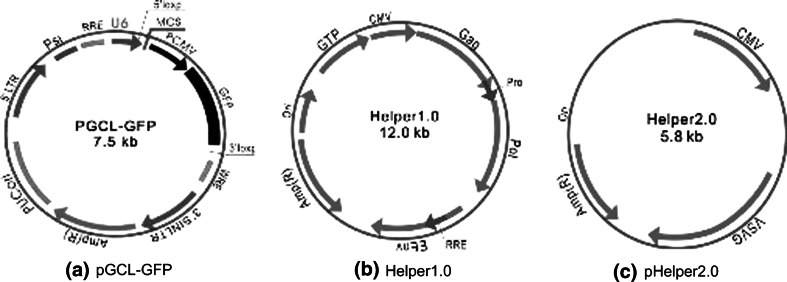
Lentiviral vectors for *pten* shRNA

The *pten* shRNA target sequences were designed by GeneChem Company (Table 1). The lentiviral vector system included three plasmids, pGCL-GFP, pHelper 1.0, and pHelper 2.0 (Fig. 1), which was extracted with the Qiagen plasmid extraction kit. The 293T cells were transfected by cationic liposomes (Lipofectamine 2000) to harvest the active virosomes, which was stored at −70 °C.

**Table 1:** *pten* shRNA target sequences

	Forward 5′ → 3′	Reverse 3′ → 5′
*pten* shRNA1	GGCGCUAUGUAUAUUAUUATT	UAAUAAUAUACAUAGCGCCTT
*pten* shRNA2	GUAUAGAGCGUGCGGAUAATT	UUAUCCGCACGCUCUAUACTT
*pten* shRNA3	GAUCUUGACCAAUGGCUAATT	UUAGCCAUUGGUCAAGAUCTT

### Transfection of shRNA lentivirus

We rapidly engineered transgenic rats by directly injecting the shRNA lentivirus into their ovaries [12]. 2 weeks later, the rats were killed to harvest their ovaries, which were directly frozen and then sliced with a thermostatic frozen slicer. The thickness of the slices was approximately 5 μm. The GFP expression of the slices was observed under fluorescence microscopy.

### Statistical analysis

All data are presented as the mean ± SE ($\overline{x} \pm s$). Analysis of variance and an LSD test between the groups were performed using SPSS (Version 10.0 Software, Tsinghua University, China). A value of *P* < 0.05 was considered statistically significant.

## Results

### The establishment of PCOS rat mode

#### Morphology and weight of ovaries of PCOS model rat

**Fig. 2:**
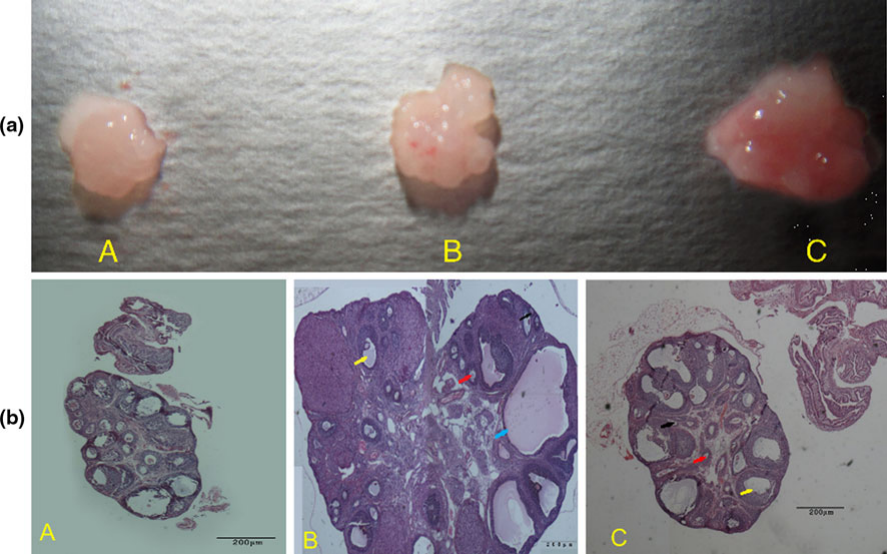
Appearances of ovaries in the control groups and the model group. **a**, **b** Indicate external ovarian appearance and tissue morphological appearances (HE ×40), respectively. *A* day 0 control, *B* day 12 control, *C* model. *Black arrow* primordial follicle, *red arrow* preantral follicle, *yellow arrow* antral follicle, *blue arrow* cystic follicle

Figure 2a, b shows that the volume of the ovaries and the number of cystic dilatation ovarian follicles have both increased. Table 2 indicates that the average weight of the ovaries in the model group treated with norethindrone and HCG was significantly higher than those in the control group.

**Table 2:** Weight of ovaries in control and model groups

Group	Sample number	Unilateral ovary weight (mg)
0 day control	12	11.42 ± 1.46
12 day control	12	20.82 ± 4.04*
Model	36	48.44 ± 8.04^##,△△^

* Showed significant difference between day 0 control and day 12 control at 0.05 level; ^##^ and ^△△^ showed significant difference between the model group and the control group at day 0 and day 12 (*P* < 0.01), respectively

### Serum hormone levels

From Table 3, we found that the levels of all hormones except hormone *E* (estradiol) were significantly higher in the model group than in the control group at both day 0 and day 12 (*P* < 0.01). There was no significant difference in the hormone levels of the control group at day 0 and day 12.

**Table 3:** Serum hormone analysis of the control group and the model group

Group	FSH (IU/L)	LH (IU/L)	*T* (ng/dL)	*E* (pg/mL)	INS (μIU/mL)	LH/FSH
0 day control	1.64 ± 0.78	0.94 ± 0.22	6.84 ± 0.34	25.78 ± 2.28	12.84 ± 2.65	0.66 ± 0.18
12 day control	1.21 ± 0.09	1.01 ± 0.17	5.49 ± 0.80	24.11 ± 1.11	13.44 ± 2.95	0.83 ± 0.08
Model	4.80 ± 0.74^##,△△^	10.93 ± 1.25^##,△△^	15.83 ± 10.44^##,△△^	28.3 ± 7.98	17.35 ± 3.51^##,△△^	2.29 ± 0.09^##,△△^

* Showed significant difference between day 0 control group and day 12 control group at 0.05 level; ^##^ and ^△△^ showed significant difference between the model group and the control group at day 0 and day 12 (*P* < 0.01), respectively

### Expression pattern of *pten* in polycystic ovary tissue

**Fig. 3:**
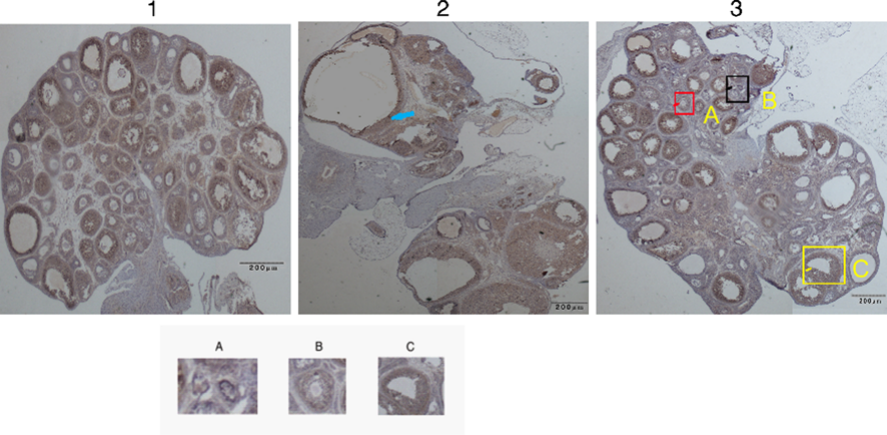
Immunohistochemical results of PTEN protein expression in the ovaries of the control and model groups (×40). *A*–*C* Indicate primordial follicles, preantral follicles, and antral follicles, respectively

Immunohistochemical results showed that *pten* protein was expressed in each follicular stage for both the control and the model groups (Fig. 3). However, the location of the expression seemed to be based on the developmental stage of the follicle. Pten protein was expressed mainly in the oocyte cytoplasm for the primordial follicles, while expressed in granulosa cells for the preantral and antral follicles, as well as the dilation and atretic follicles.

**Fig. 4:**
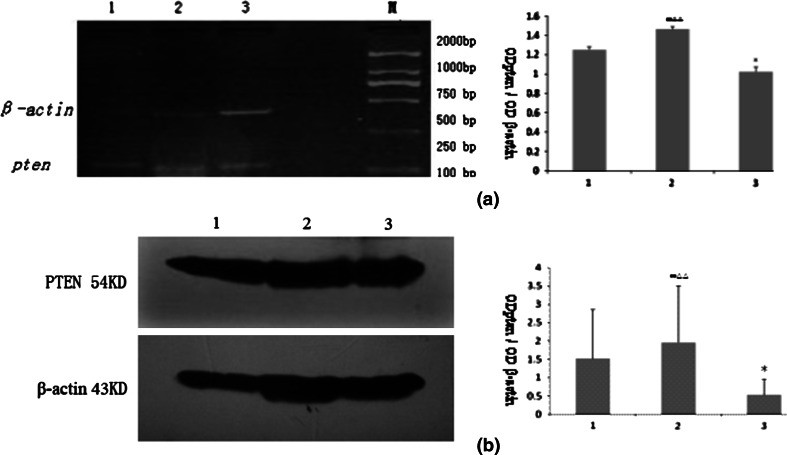
Expressions of PTEN in the ovaries of the control and model groups. **a**, **b** Indicate the pten mRNA expression revealed by RT-PCR ($\overline{x}$ ± *s*, *n* = 3) and Expressions of PTEN protein by western-blot, respectively. *1*, *2*, and *3* represent day 0 control, model, and day 12 control, respectively

Semi-quantitative analyses of RT-PCR and Western blot revealed that the expression of pten mRNA and PTEN protein in the day 0 control was significantly higher than in the day 12 control (*P* < 0.05), while the expression of pten in the model was significantly higher than those in both the day 0 control and day 12 control (*P* < 0.01) (see Fig. 4a, b).

### Formation of the polycystic ovary by the transfection with shRNA lentivirus

**Fig. 5:**
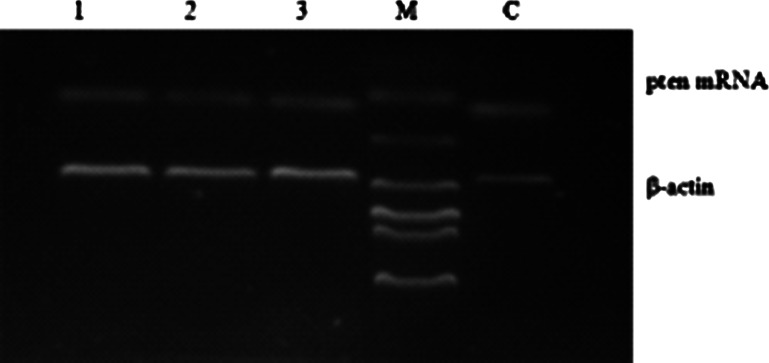
Changes of pten mRNA levels in ovaries after pten-shRNA1, pten-shRNA2, and pten-shRNA3 interference by RT-PCR. *M* marker, *C* control, *1* pten-shRNA1, *2* pten-shRNA2, *3* pten-shRNA3

**Fig. 6:**
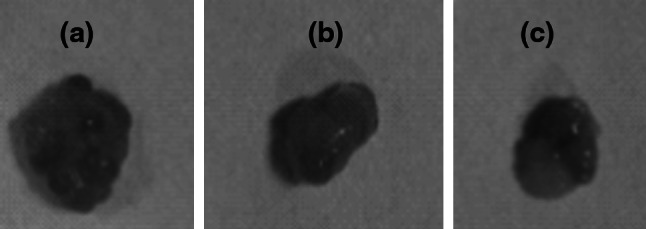
Changes of polycystic ovarian morphology after pten shRNA lentivirus transfection. *A*–*C* showed the model control group, the empty vector + model group and the pten shRNA + model group, respectively

**Fig. 7:**
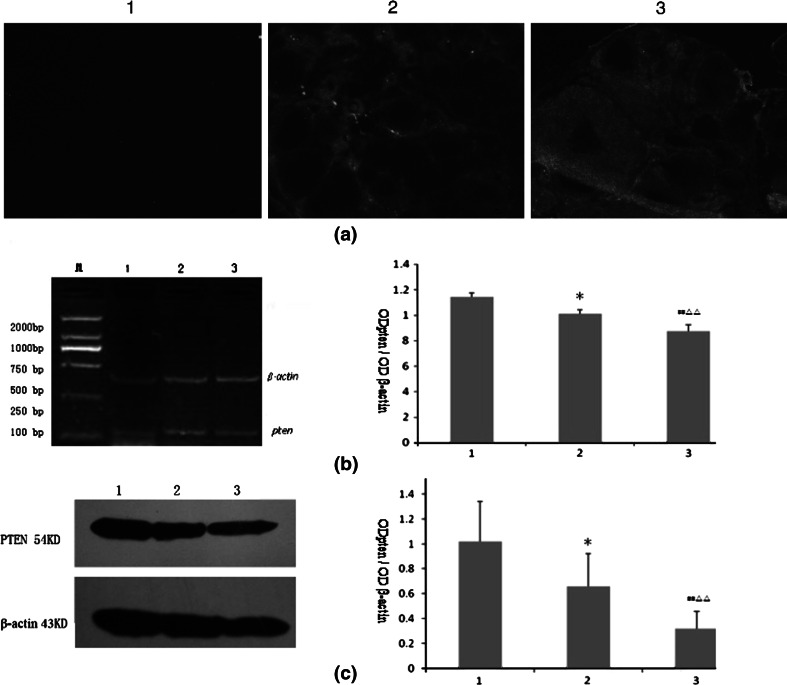
Expressions of PTEN after transfecting the pten shRNA lentivirus into the ovaries of the control and model groups. *1*, *2*, and *3* represent day 0 control, model, and day 12 control, respectively. **a** Expression of GFP in ovaries after pten shRNA lentivirus transfection (×40). **b** Expressions of pten mRNA revealed by RT-PCR. **c** PTEN protein expression revealed by western-blot ($\overline{x}$ ± *s*, *n* = 3)

**Fig. 8:**
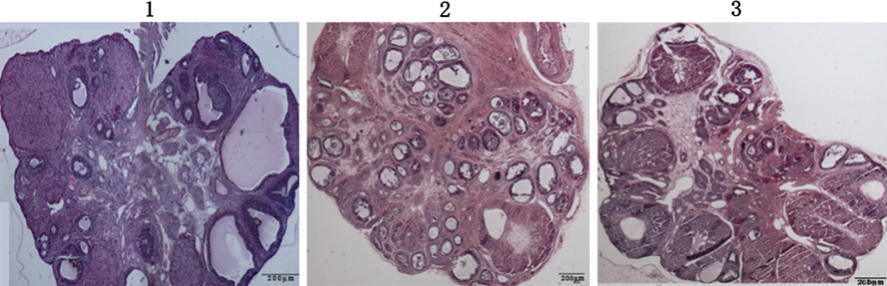
Morphological observations of ovarian tissue after pten shRNA lentivirus transfection (HE ×40). Note: *1*, *2* and *3* referred to the model control group, the empty vector + model group and the pten shRNA + model group, respectively

After the transfection of the lentiviral vectors carrying *pten* shRNA into the in vivo ovaries of each group (Fig. 5), ovarian size and weight in the lentivirus *pten* shRNA + model group decreased markedly (Table 4; Fig. 6). GFP was expressed in the *pten* shRNA + model group and in the blank vector + model group, when we viewed the frozen ovarian sections under a fluorescent microscope 12 days after transfection. Semi-quantitative analysis of RT-PCR and Western blot showed that the expression of pten mRNA and PTEN protein in the *pten* shRNA + model group was significantly lower than the expression of the model and blank vector + model groups (*P* < 0.01) (Fig. 7a–c). Serum hormone analysis demonstrated that only the *T* hormone of the *pten* shRNA + model was significantly higher than those of the model control and the empty vector + model at the 0.05 and 0.01 levels, respectively (Table 4). In addition, HE staining results revealed that cystic dilatation of the follicles and atretic follicles was obviously reduced (Fig. 8).

**Table 4:** Changes of polycystic ovarian weight and serum hormone analysis after *pten* shRNA lentivirus transfection

Group	One ovary weight (mg)	FSH (IU/L)	LH (IU/L)	*T* (ng/dL)	INS (μIU/mL)
Model control	50.02 ± 2.5	4.60 ± 0.58	9.55 ± 1.50	14.95 ± 5.54	17.06 ± 2.55
The empty vector + model	41.42 ± 1.24*	4.72 ± 1.09	8.87 ± 2.07	12.33 ± 3.73	16.44 ± 3.07
*pten* shRNA + model group	30.26 ± 1.04^##,△△^	4.42 ± 0.60	9.03 ± 2.25	5.18 ± 2.69^##,△△^	16.35 ± 3.61

* Showed significant difference between 0 day control group and 12 day control group at 0.05 level; ^##^ and ^△△^ showed significant difference of model group with control group at 0 and 12 day (*P* < 0.01), respectively

## Discussion

### Importance of constructing PCOS rat models

Due to the difficulties of acquirement of clinical patient samples, the construction of PCOS animal models seemed to be very important to the study of the pathogenic mechanisms of PCOS. In 1989, Bogoviol et al. [10] established the norethindrone + HCG modeling method. In accordance with this method, we constructed the PCOS rat model used in this study, and we found that size and weight for ovarian of the model group was significantly higher than those of the control groups (*P* < 0.01). An increased cystic dilatation of follicles was observed on the surface of the ovaries in the model group. HE staining results showed that the number of the primordial follicles was significantly lower in the model group than in the day 12 control group (*P* <0.01), while the number of preantral follicles was significantly higher (*P* < 0.01). The level of antral follicles in the model group was significantly reduced (*P* < 0.01) from the day 12 control group, and in the model group, the cystic dilatation of follicles and atretic follicles was increased significantly (*P* < 0.01). In addition, in the model group, fewer granular layers were identified, and the granulosa cells were arranged loosely, or even lost. In addition, the results of serum hormone detection showed that levels of LH, T, INS, and LH/FSH increased significantly (*P* < 0.01) in the model group. These results further demonstrate the importance of constructing PCOS animal models and show that norethindrone + HCG modeling is a satisfactory technique. Furthermore, steps of constructing this PCOS model is simple, and experimental period is short. Maybe, the norethindrone + HCG model is the good model for PCOS research in rat.

There are many methods of building PCOS rat models, such as using dehydroepiandrosteron subcutaneous injection [13], letrozole gavage [14] and by exposing rats to continuous light [15], etc. But these models imitate one or a few aspects of the pathological characteristic of PCOS. Perhaps, there is no model that can be fully reflected the characteristics of PCOS. Therefore, when considering the choice of a model, according to the research purpose, choosing the Suitable rat model is the best solution.

### Expression of *pten* in polycystic ovarian follicles

Different patterns of *pten* expression in polycystic ovaries have been reported in different animals at different growth stages. For example, Ding et al. [16] reported that PTEN protein was found only in the cytoplasm of the pig oocyte. Goto et al. [17] observed that the expression of PTEN protein in the granular cell increased as the human ovarian follicle grew into its later stages.

In the present study, our immunohistochemical results showed that *pten* could be expressed in all stages of the follicles (Fig. 3); however, *pten* expression was mainly found in the oocyte cytoplasm of primordial follicles and in the granulosa cells of preantral follicles and antral follicles, as well as dilation and atretic follicles. These results indicate that the spatio-temporal expression of *pten* varies according to the development of the rat ovarian follicles. Furthermore, semi-quantitative analysis of RT-PCR and Western blot revealed that the expression of *pten* mRNA and PTEN protein decreased significantly as the ovary grew in the control group but increased significantly (Fig. 4a, b) in the model group, suggesting that PTEN protein might play an important role in the pathogenesis of PCOS [18]. Others, in Fig. 7a–c the expression of pten mRNA and PTEN protein in the blank vector + model group is significantly lower than the expression of the model group (*P* < 0.01). Perhaps, just because some normal saline was injected in the blank vector + model group, while normal saline was not injected in the model group, and the normal saline may dilute the concentration of testosterone, thereby reducing related to PCOS symptoms in the blank vector + model group.

### Roles of *pten* in the development of polycystic ovarian follicles

The *pten* gene plays a key role in the initiation, development, apoptosis, and atresia of the primordial follicle. For example, by knocking down and silencing *pten*, Li et al. [19] found that a previously activated ovarian follicle could develop to demonstrate the capacity for fertilization after being cultured outside the body. Moreover, Fan et al. [8] reported that rats lacking *pten* expression in granular cells (conditional knockdowns) demonstrated a higher frequency of ovulation and greater number of offspring. These results suggest that in the early growth stages of the ovarian follicle, PTEN protein can insure that the primordial follicle remains quiescent, while in later growth stages of the ovarian follicle, PTEN protein can regulate not only the proliferation and apoptosis of granular cells but also the development and atresia of the sinusoid ovarian follicle.

In the present research, we have found that the expression of pten mRNA and PTEN protein increased significantly in the PCOS model rat group. Pten shRNA effectively reduced the severity of PCOS, resulting in a lower ovarian volume and weight, a smaller quantity of preantral follicles, cystic dilatation, and atretic follicles. Moreover, we found that the content of *T* hormone was also obviously reduced, which could also interfere the pathogenesis of PCOS. Therefore, we suggest that: (1) PTEN plays an important role in the pathogenesis of PCOS; (2) a high level expression of PTEN in the PCOS can induce apoptosis of the preantral follicle and sinusoid follicle; (3) the final result may be the arrested development of the ovarian follicle and anovulia.

Moreover, PTEN has also been reported to be an important signal molecule in the pathway of PTEN-PI3K-AKT, which is closely correlated with the pathogenesis of PCOS. In addition, previous research suggested that transfecting pten into an ovarian cancer cell might be a new interventional method for curing ovarian cancer. The results of the present study also indicate that pten might be a new target gene to treat PCOS; however, the specific functional pathway relating pten and the apoptosis of polycystic ovarian cells remains to be explored.
